# Low Exposures to Amphibole or Serpentine Asbestos in Germline *Bap1*-mutant Mice Induce Mesothelioma Characterized by an Immunosuppressive Tumor Microenvironment

**DOI:** 10.1158/2767-9764.CRC-23-0423

**Published:** 2024-04-08

**Authors:** Yuwaraj Kadariya, Eleonora Sementino, Maggie Ruan, Mitchell Cheung, Parham Hadikhani, Hatice U. Osmanbeyoglu, Andres J. Klein-Szanto, Kathy Cai, Joseph R. Testa

**Affiliations:** 1 Cancer Prevention and Control Program, Fox Chase Cancer Center, Philadelphia, Pennsylvania.; 2 Department of Biomedical Informatics, School of Medicine, University of Pittsburgh, Pittsburgh, Pennsylvania.; 3 UPMC Hillman Cancer Center, Cancer Biology Program, School of Medicine, University of Pittsburgh, Pittsburgh, Pennsylvania.; 4 Histopathology Facility, Fox Chase Cancer Center, Philadelphia, Pennsylvania.

## Abstract

Asbestos and *BAP1* germline mutations are risk factors for malignant mesothelioma (MM). While it is well accepted that amphibole asbestos is carcinogenic, the role of serpentine (chrysotile) asbestos in MM has been debated. To address this controversy, we assessed whether minimal exposure to chrysotile could significantly increase the incidence and rate of MM onset in germline *Bap1*-mutant mice. With either crocidolite or chrysotile, and at each dose tested, MMs occurred at a significantly higher rate and earlier onset time in *Bap1*-mutant mice than in wild-type littermates. To explore the role of gene–environment interactions in MMs from *Bap1*-mutant mice, we investigated proinflammatory and protumorigenic factors and the tumor immune microenvironment (TIME). IHC and immunofluorescence staining showed an increased number of macrophages in granulomatous lesions and MMs. The relative number of CD163-positive (CD163^+^) M2 macrophages in chrysotile-induced MMs was consistently greater than in crocidolite-induced MMs, suggesting that chrysotile induces a more profound immunosuppressive response that creates favorable conditions for evading immune surveillance. MMs from *Bap1*-mutant mice showed upregulation of CD39/CD73-adenosine and C-C motif chemokine ligand 2 (Ccl2)/C-C motif chemokine receptor 2 (Ccr2) pathways, which together with upregulation of IL6 and IL10, promoted an immunosuppressive TIME, partly by attracting M2 macrophages. Interrogation of published human MM RNA sequencing (RNA-seq) data implicated these same immunosuppressive pathways and connections with CD163^+^ M2 macrophages. These findings indicate that increased M2 macrophages, along with upregulated CD39/CD73-adenosine and Ccl2/Ccr2 pathways, contribute to an immunosuppressive TIME in chrysotile-induced MMs of *Bap1*-mutant mice, suggesting that immunotherapeutic strategies targeting protumorigenic immune pathways could be beneficial in human *BAP1* mutation carriers who develop MM.

**Significance:**

We show that germline *Bap1*-mutant mice have enhanced susceptibility to MM upon minimal exposure to chrysotile asbestos, not only amphibole fibers. Chrysotile induced a more profound immune tumor response than crocidolite in *Bap1*-mutant mice by upregulating CD39/CD73-adenosine and Ccl2/Ccr2 pathways and recruiting more M2 macrophages, which together contributed to an immunosuppressive tumor microenvironment. Interrogation of human MM RNA-seq data revealed interconnected immunosuppressive pathways consistent with our mouse findings.

## Introduction

Malignant mesothelioma (MM) is an incurable cancer causally associated with asbestos (1). This inflammation-associated malignancy develops in the mesothelial lining, most often in the pleura and peritoneum, with a latency period of several decades from the initial exposure to asbestos.

Asbestos consists of serpentine (chrysotile) and amphibole minerals. Serpentine fibers have a curved, wavy morphology. In contrast, the amphibole group includes fibers such as crocidolite, which are firm and linear. Amphibole fibers are thought to be much more carcinogenic than chrysotile (2). This is due to the fact that chrysotile is biodegradable and most of it is cleared from the upper respiratory tract, as well as because it has a lower toxicity potential compared with amphibole (3, 4). Notably, the iron content of chrysotile is 1 order of magnitude lower than that of crocidolite, but acellular *in vitro* studies have revealed that chrysotile has a much faster dissolution rate that prompts greater release of available active surface iron and certain other metals, which may promote the formation of toxic hydroxyl radicals. Hence, some investigators have concluded that the toxicity paradigm of non-biodurable fibers such as chrysotile should also consider the release of toxic metals such as chromium, manganese, and nickel into the environment during the rapid dissolution process (4). While the role of chrysotile in MM has been controversial, in 2021, the Environmental Protection Agency’s final risk evaluation for chrysotile identified several health risks to workers and others exposed to this mineral, including MM and lung cancer (5).

In addition to asbestos, genetic factors have emerged as risk factors for MM development. In particular, there is overwhelming evidence that germline mutations in *BAP1* predispose carriers to MM and certain other benign and malignant tumors (6–9). In 2011, we reported germline *BAP1* mutations in two families with multiple MMs (6). Interestingly, chrysotile asbestos was detected in five of the five homes in which all the affected members of one family had lived. Traces of chrysotile and tremolite asbestos were found in the home where all affected members of the second family were raised. In that report, we noted that living in an asbestos-containing home is generally thought to be associated with modest levels of exposure, as observed in people who have not been occupationally exposed, a group that represents a growing fraction of those with MM (6). Similar to human *BAP1* mutation carriers, heterozygous *Bap1*-mutant mice develop a spectrum of spontaneous malignant tumors, including rare MMs (10). Underscoring the relevance of *BAP1* inactivation in MM pathogenesis, mice with heterozygous *Bap1* knockout or knock-in mutations exhibit a markedly higher incidence and accelerated onset of crocidolite-induced MM compared with genetically normal (wild-type, WT) littermates (10, 11). This was later shown to be true even with minimal doses of crocidolite (12). However, whether chrysotile has a similar effect has not yet been tested. We hypothesized that germline *Bap1* heterozygous mutant mice would be highly susceptible to serpentine asbestos, even low doses of these fibers, and that their predisposition might be associated with an altered inflammatory immune response. In this report, we demonstrate that low concentrations of both crocidolite and chrysotile can induce MM in mice with germline *Bap1* heterozygous mutations. We also demonstrate that the relative number of CD163-positive (CD163^+^) M2 macrophages in chrysotile-induced MMs is considerably greater than in crocidolite-induced tumors, suggesting that chrysotile induces a more profound immunosuppressive tumor microenvironment that creates favorable conditions for tumors to evade immune surveillance in *Bap1^+/^^−^* mice.

## Materials and Methods

### Heterozygous *Bap1*-mutant Mice

Mouse experiments were carried out at different times over a 4-year period. All experiments were performed using germline *Bap1* heterozygous knockout mice and WT littermates. For three asbestos experiments, we used a germline *Bap1* heterozygous model in which the knockout allele has a deletion of exons 6 and 7, which results in a frameshift and predicted premature truncation of the Bap1 protein; genotyping of these mice has been reported elsewhere (11). The net effect was similar to that observed in a BAP1 tumor predisposition syndrome (BAP1-TPDS) family with an intron 6 splice site mutation in *BAP1*, resulting in the loss of exon 7 (6). Another experiment, using crocidolite at a very low dose (total, 0.1 mg/mouse), was performed using a germline *Bap1* heterozygous knock-in model carrying a nonsense mutation in exon 16 (*Bap1^+/^^L^*; ref. 10), a mutation identical to that observed in another BAP1-TPDS family (6). Genotyping of these mice was performed as described previously (10). Both mouse models have an FVB genetic background and develop MM with virtually identical incidences and onset times when injected with crocidolite asbestos at our standard dose of 3.2 mg/mouse (10, 11). Because the mutations in each mouse model are predicted to result in a truncation of the Bap1 protein, both are referred to here as *Bap1^+/^^−^* mice.

All mouse studies were performed in accordance with NIH's Guide for the Care and Use of Laboratory Animals. The Fox Chase Cancer Center (FCCC) Committee on the Ethics of Animal Experiments approved the study protocol using asbestos.

### Asbestos Injections

To assess the susceptibility of *Bap1*^+/−^ mice to the carcinogenic effects of asbestos, *Bap1^+/^^−^* and *Bap1^+/+^* (WT) littermates at 8–10 weeks of age were injected intraperitoneally with crocidolite or chrysotile (Union for International Cancer Control standards, SPI Supplies) in Hank's Balanced Salt Solution according to our standard method (10, 13). Note that it was not the intention of this study to directly compare the carcinogenicity of crocidolite and chrysotile; therefore, the low doses used here were not identical for these two forms of asbestos. For crocidolite, the animals were injected with 0.1 or 0.0125 mg of fibers every 21 days × 8 injections (totals, 0.8 mg/mouse and 0.1 mg/mouse, respectively). For chrysotile, mice were injected with 0.05 mg of fibers every 21 days × 8 injections (total, 0.4 mg/mouse). Because we had never previously tested the carcinogenicity of chrysotile in mice, we first assessed its ability to induce MM when injected at our standard dose of 0.4 mg every 21 days × 8 (total, 3.2 mg/mouse).

All mice were examined daily and sacrificed by CO_2_ asphyxiation followed by cervical dislocation based on evidence of labored breathing, severe weight loss (>10% of body weight), abdominal bloating, lethargic behavior, hunched back, and/or difficulty in walking, or when tumor burden was obvious, in accordance with a protocol approved by the FCCC Institutional Animal Care and Use Committee. At necropsy, all organs were histopathologically examined for evidence of tumor lesions or overt malignancy. To evaluate tumor invasiveness and spreading, complete necropsies were performed on all mice by examining the thoracic, abdominal, and pelvic cavities for tissue collection. Tumor tissues and other organs were collected in 10% buffered formaldehyde, fixed for 24–48 hours, and processed for histopathologic examination. Tumor tissues and ascitic fluids were collected and stored at −80°C for further analysis.

### Histopathologic Staining

To investigate the proinflammatory and tumor immune microenvironment (TIME) of MMs from *Bap1*-mutant mice treated with asbestos, we selected seven to eight tumors from each of the two groups: (i) *Bap1^+/^^−^* (crocidolite), and (ii) *Bap1^+/^^−^* (chrysotile). Each tumor was subjected to hematoxylin and eosin (H&E), IHC, and immunofluorescence (IF) staining analysis.

Fixed mouse tumor tissues were dehydrated and embedded in paraffin. Serial sections of formalin-fixed/paraffin-embedded tumor specimens were mounted on positively charged microscope slides. H&E-stained sections were used for morphologic evaluation, and unstained sections were used for IHC staining with the antibodies listed in Supplementary Table S1, according to the manufacturers’ instructions.

IHC was performed on a Ventana Discovery XT automated staining instrument (Ventana Medical Systems) using Ventana reagents according to the manufacturer's protocol. Immune complexes were detected using the Ventana OmniMap anti-Rabbit detection kit (760-4311) and developed using the Ventana ChromMap DAB detection kit (760-159). Slides were then counterstained with hematoxylin II (#790-2208) for 8 minutes, followed by Bluing reagent (#760-2037) for 4 minutes. Slides were then mounted and photographed using a Nikon Eclipse 50i microscope, as described previously (14).

For IF, fluorescent 3‐plex staining was performed using the U Discovery 3‐Plex RUO staining procedure, which utilizes tyramide signal amplification. The assay was run on the Ventana automated staining platform (Roche Tissue Diagnostics) using Ventana /Roche reagents, unless otherwise noted. The multiplex protocol involves deparaffinization (EZ‐Prep solution, Ventana, 950‐102) and antigen retrieval (DISCOVERY CC1 solution; 950‐500) at high temperature followed by three sequential rounds of (i) incubation of the target antigen with a primary antibody, (ii) binding of the primary antibody to horseradish peroxidase (HRP)‐conjugated secondary antibody, (iii) enzymatic reaction of HRP in the primary‐secondary antibody complex with the tyramide‐fluorophore detection system, and (iv) covalent binding of the tyramide‐fluorophore complex to the tissue. The primary antibodies used are listed in Supplementary Table S1. Fluorescence detection was performed using the DISCOVERY FAM Kit (760‐243), DISCOVERY Cy5 Kit (760‐238), and DISCOVERY Rhodamine 6G Kit (760‐244). After completion of the three staining rounds, the sections were counterstained with quantum dot‐4′,6‐diamidino‐2‐phenylindole (DISCOVERY, #760‐4196). A Ventana ChromMap DAB detection kit (760–159) was used according to the manufacturer's instructions. As a negative control, the primary antibody was replaced with normal rabbit IgG to confirm the absence of specific staining.

Quantitative image analysis was performed using an Aperio ScanScope CS 5 slide scanner. The scanned images were viewed using ImageScope, version 11.1.2.760 (Aperio). The positive percentage scores for CD206 and CD163 were quantified using the Aperio V9 algorithm.

### ELISA

Ascitic fluids from five *Bap^+/+^* mice and four *Bap1^+/^^−^* mice that developed chrysotile-induced MMs were collected at the time of euthanization. The samples were centrifuged at 13,000 rpm at 4°C followed by storage at −80°C. Prior to performing an ELISA, samples were thawed on ice, and the analysis was carried out using a commercially available mouse IL6 Quantikine ELISA kit (R&D Systems, M6000B) and mouse IL10 ELISA Kit (Abcam, ab255729), according to the manufacturer's instructions. All samples were assayed in triplicate.

### Human MM Cell Lines and Cell Culture

Most of the pleural MM cell lines used in this study were generated as reported previously (15). Other MM lines included NCI-H2452 (catalog no. CRL-5946; RRID:CVCL_1553) and MSTO-211H (catalog no. CRL-2081; RRID:CVCL_1430), which were obtained from the ATCC. All MM cell lines were periodically screened for *Mycoplasma* by our Cell Culture Facility by transferring supernatant from cell cultures to indicator cultures of Vero kidney cells and then staining for cytoplasmic DNA (*Mycoplasma*) using Hoechst dye and fluorescence microscopy. All cell lines tested were negative for *Mycoplasma* contamination. Cells were maintained in RPMI1640 medium supplemented with 10% FBS containing 100 µg/mL penicillin and streptomycin and 2 mmol/L l-glutamine.

### NCI Human MM Dataset

We downloaded MM normalized RNA sequencing (RNA-seq; *n* = 100), germline mutation status, and clinical data from Supplementary Tables S1, S3, and S4 of the NCI report by Nair and colleagues (16). The RNA-seq data were normalized using trimmed mean of M-values, and log counts per million transformed using edgeR (17). Student *t* test was used for the analysis in Fig. 7A, and Spearman rank correlation was used for the analysis in Fig. 7C.

### Immunoblotting

Western blots were prepared with 30 µg of protein per sample, as described elsewhere (18). The primary antibodies used are listed in Supplementary Table S1. Appropriate secondary antibody, anti-rabbit IgG, HRP-linked Antibody (#7074 Cell Signaling Technology), was used at 1:5,000 dilution except for vinculin, which was at 1:50,000.

### Data Availability Statement

All relevant data are within the article and its Supporting Data files.

## Results

### Mice with Heterozygous Germline Mutations of *Bap1* are Markedly Susceptible to MM Upon Minimal Exposure to Either Crocidolite or Chrysotile Asbestos

MMs developing in *Bap1^+/^^−^* and *Bap1^+/+^* littermates injected with chrysotile or crocidolite typically were diffuse peritoneal lesions sometimes accompanied by ascites. An anatomic image of a chrysotile-induced MM in a *Bap1^+/^^−^* mouse is shown in Supplementary Fig. S1.

**FIGURE 1:**
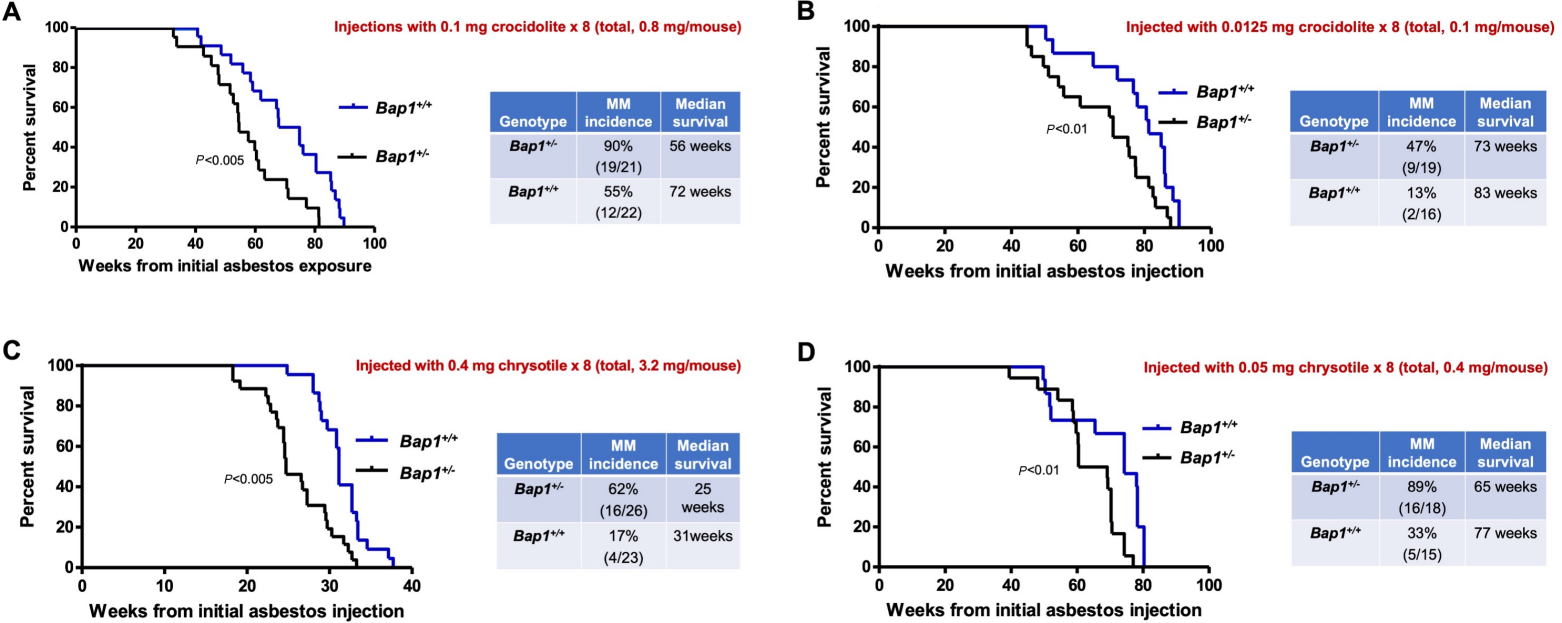
MM formation induced by crocidolite or chrysotile asbestos in mice with germline *Bap1* heterozygous mutation. MM formation induced by minimal exposure to crocidolite (**A** and **B**). **A,***Bap1^+/−^* and *Bap1^+/+^* (WT) littermates injected with a total of 0.8 mg of crocidolite fibers. Left, Kaplan–Meier curves depicting survivals in *Bap1^+/−^* and *Bap1^+/+^* groups. Right, Summary of MM incidence and median survival times. **B,** Results for *Bap1^+/−^* and *Bap1^+/+^* mice injected with a total of 0.1 mg of crocidolite. Left, Kaplan–Meier curves. Right, Summary of MM incidence and median survival times. MM formation induced by high (**C**) and minimal (**D**) exposure to chrysotile. **C,***Bap1^+/−^* and *Bap1^+/+^* mice were injected with a total of 3.2 mg of chrysotile. Left, Kaplan–Meier survival curves. Right, MM incidence and median survival times. **D,***Bap1^+/−^* and *Bap1^+/+^* mice injected with a total of 0.4 mg/mouse of crocidolite. Left, Kaplan–Meier curves. Right, MM incidence and median survival times. For all Kaplan–Meier survival curves, deaths due to all causes are included. Some mice (especially WT animals) succumbed because of adhesion-related intestinal obstructions and/or liver fibrosis.

With a total dose of 0.8 mg/mouse of crocidolite, *Bap1^+/^^−^* mice succumbed to disease much earlier than their WT littermates, with an overall median survival of 56 weeks from the time of the first asbestos injection in *Bap1*-mutant mice compared with 72 weeks in *Bap1^+/+^* (WT) littermates (Fig. 1A), which was highly significant (*P* < 0.005, log-rank test). Peritoneal MMs were observed in 19 of 21 (90%) *Bap1^+/^^−^* mice versus 12 of 22 (55%) WT animals (*P* < 0.01, Fisher exact test). At a lower total dose of 0.1 mg/mouse of crocidolite, the median survival was 73 weeks in *Bap1*-mutant mice and 83 weeks in WT mice (*P* < 0.01, log-rank test), and the incidence of MM was 47% (9/19 mice) in *Bap1^+/^^−^* mice compared with only 13% (2/16) in *Bap1^+/+^* mice (Fisher exact test, *P* < 0.03; Fig. 1B).

As mentioned earlier, for chrysotile, we first tested a relatively high dose, equivalent to what we have used as a “standard” in the past for crocidolite (i.e., total = 3.2 mg/mouse; refs. 10, 11, 13, 18). At this dose, *Bap1*-mutant mice died earlier than similarly treated WT mice, with a median survival of 25 weeks in *Bap1*^+/−^ mice compared with 31 weeks in WT littermates (*P* < 0.005, log-rank test; Fig. 1C). Notably, the survival of chrysotile-exposed *Bap1*^+/−^ and *Bap1*^+/+^ mice was much shorter than that reported in our previous studies using an identical dose and dosing regimen for crocidolite (10, 11). Adhesion-related intestinal obstructions and liver fibrosis were seen in both WT and *Bap1^+/^^−^* mice, irrespective of whether they developed MM. Many WT animals, but also some *Bap1^+/^^−^* mice, died of such complications. Histologic examples of asbestos-related liver fibrosis and adhesions are shown in Supplementary Fig. S2, with a summary of the ratio of animals that developed these pathologies in all cohorts presented in Supplementary Table S2. The incidence of chrysotile-induced MMs was 62% (16/26) in *Bap1^+/^^−^* mice compared with only 17% (4/23) in *Bap1*^+/+^ mice. The incidence of MMs induced by chrysotile in WT mice was considerably less than the 32%–35% MMs induced by crocidolite in WT mice in our earlier studies (10, 11). At a much lower total dose of chrysotile (0.4 mg/mouse), peritoneal MMs were unexpectedly identified in a higher percentage of mice than at a higher dose, although the time to tumor onset was much longer. MMs were observed in 16 of 18 (89%) *Bap1^+/^^−^* mice compared with 5 of 15 (33%) WT mice (*P* < 0.001, Fisher exact test). The median survival was 65 weeks in *Bap1^+/^^−^* mice versus 77 weeks in WT mice (Fig. 1D; *P* < 0.01, log-rank test).

### Histopathologic Assessment of MMs Induced by Crocidolite or Chrysotile in *Bap1*-mutant Mice Show a Biphasic Phenotype

Pathologic diagnosis of crocidolite- and chrysotile-induced MMs was performed independently by histopathologic examination of H&E-stained tumor sections by two experimental pathologists (K. Cai, A.J. Klein-Szanto). The final diagnosis of MM was confirmed by IHC staining for podoplanin and WT1 (Supplementary Fig. S3). MM histology was mostly biphasic, with sarcomatoid morphology predominating, in both crocidolite-induced and chrysotile-induced tumors.

### Immune Cell Marker Studies Demonstrate That the TIME of Asbestos-induced MMs of *Bap1*-mutant Mice Contain Few T, B, and Natural Killer Cells, Whereas Tumor-associated Macrophages are Abundant, with More CD163^+^ M2 Macrophages in Chrysotile-induced Than Crocidolite-induced Tumors

**FIGURE 2:**
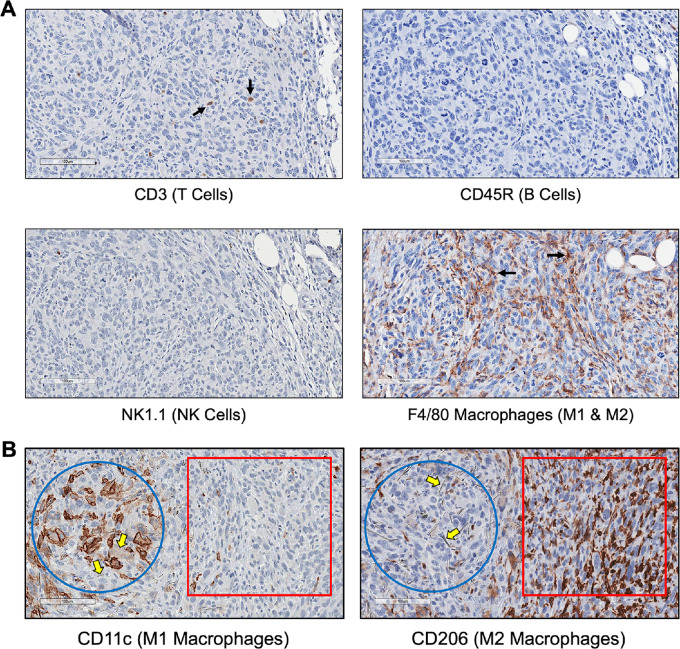
TIME of crocidolite-induced MMs from *Bap1^+/−^* mice. **A,** Serial sections of a crocidolite-induced MM from *Bap1-*mutant mouse stained with several different immune cell markers: CD3 (T cells; arrows); CD45R (B cells); F4/80 (macrophages; arrows); and NK1.1 (natural killer cells), clockwise starting from top left. **B,** Serial sections showing distribution of macrophages in granuloma and TIME of MM from *Bap1^+/−^* mouse exposed to crocidolite. Left*,* Proinflammatory CD11c-positive M1 macrophages. Right*,* Protumorigenic CD206-positive M2 macrophages. Blue circles, granulomatous lesions; red squares, MM tumor areas. Note numerous crocidolite fibers (yellow block arrows) in granulomatous lesions.

To compare the TIME of MMs induced by crocidolite versus that induced by chrysotile in *Bap1*-mutant mice, we used CD3 (T-cell marker), CD45R (B-cell marker), NK1.1 [natural killer (NK)-cell marker], and several different macrophage markers. Very few cells were positive for T-cell, B-cell, or NK-cell markers in either crocidolite-induced or chrysotile-induced MMs. However, in both crocidolite- and chrysotile-induced MMs, many cells in and around the tumor stained with the macrophage marker F4/80 (Fig. 2A). This marker detects all macrophages residing in chronically inflamed tissues and MM. Very similar results were seen in chrysotile-induced MMs from *Bap1^+/−^* mice (Supplementary Fig. S4).

Macrophages are recruited to asbestos-induced lesions by the surrounding microenvironment and produce cytokines and chemokines. After recruitment, macrophages can differentiate into distinct populations of tumor-associated macrophages (TAM): type 1 (M1) and type 2 (M2). M1 macrophages are proinflammatory and stain positively for the marker CD11c. Abundant numbers of M1 macrophages were present in granulomas, which result from chronic inflammation induced by asbestos fibers (Fig. 2B). Notably, we also observed many asbestos fibers within the granulomas. In contrast, few M1 macrophages were present in adjacent MM tumor areas.

**FIGURE 3:**
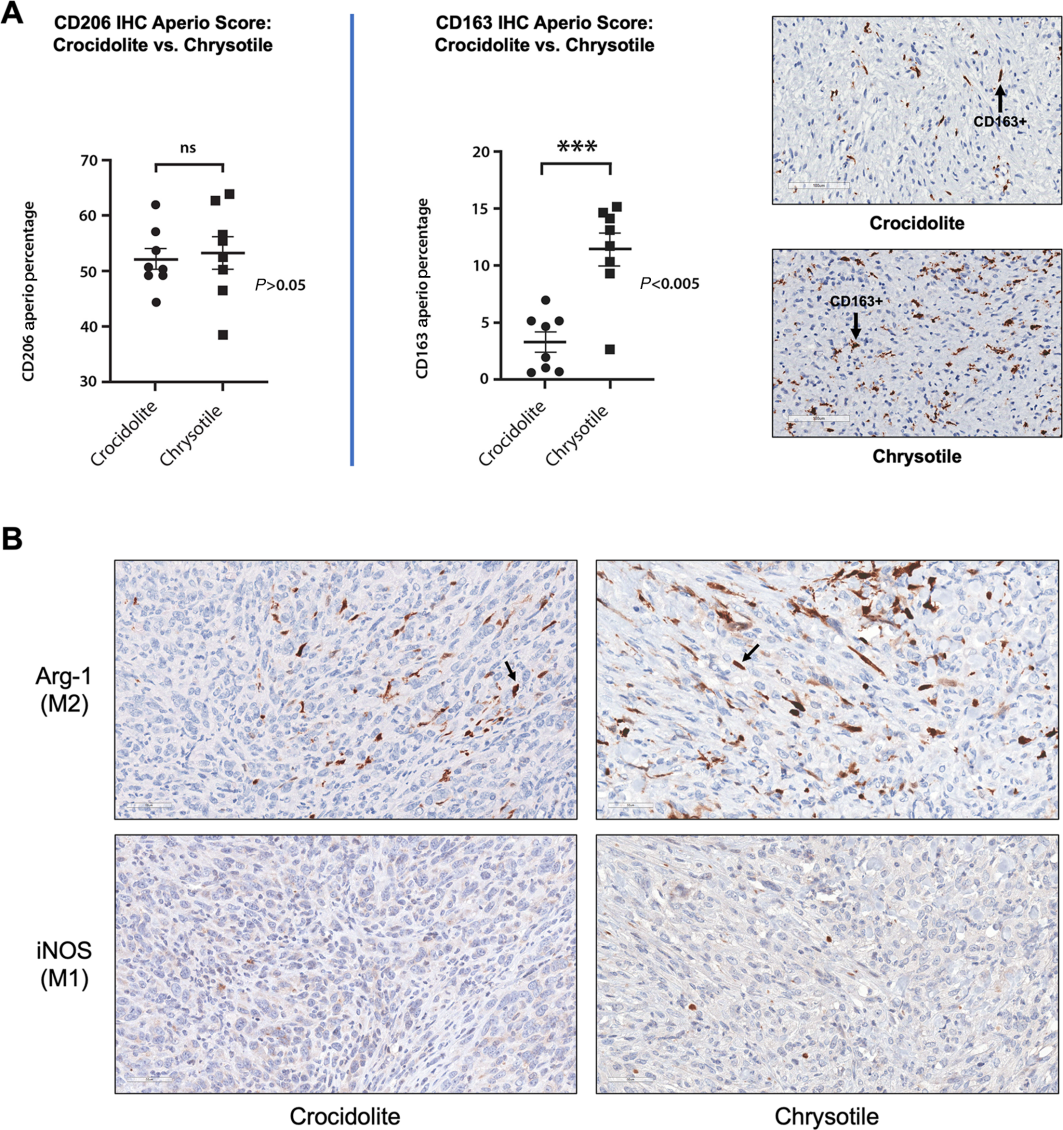
Quantification of M2 macrophages in the TIME of asbestos-induced MMs from *Bap1^+/−^* mice using an Aperio scanner, and IHC depicting arginase and iNOS staining of MM from asbestos-treated *Bap1^+/^^−^* mice. **A,** Dot plot showing percentages of CD206-stained (left) and CD163-stained (center) cells in representative histologic images from crocidolite-induced and chrysotile-induced MMs from *Bap1^+/^^−^* mice. Right, CD163 IHC staining of representative MMs from crocidolite-induced and chrysotile-induced MMs. Arrows indicate examples of cells positively stained for CD163. **B,** Serial sections of a crocidolite-induced and chrysotile-induced MMs from *Bap1^+/^^−^* mice stained for arginase (Arg-1) and iNOS. Arrows indicate Arg-1-stained M2 macrophages.

M2 polarized macrophages are protumorigenic and support cancer invasion and progression by promoting angiogenesis and immunosuppression. A subset of the M2 macrophages stained positively for the marker CD206 and a subset was CD163^+^. In our study, more CD11c-positive macrophages (M1) were found mainly in granulomas, whereas protumorigenic CD206-positive M2 macrophages were more prominent in the TIME of MM tumors but mostly absent in nearby granulomas (Fig. 2B). Notably, we observed no difference in the number of CD11c- and CD206-positive macrophages in TIMEs induced by crocidolite as compared with chrysotile; however, there was a significantly higher percentage of a very distinct subpopulation of CD163^+^ M2 macrophages in chrysotile-induced MMs than in crocidolite-induced MMs (Fig. 3A). This is notable, because CD163 is a marker specific for a tumorigenic subset of M2 macrophages that possesses a highly immunosuppressive, anti-inflammatory phenotype, and is associated with poor prognosis. No significant difference (*P* > 0.05, Fisher exact test) was observed between the percentage of CD206-stained cells in the crocidolite- and the chrysotile-induced MMs, whereas the TIME of chrysotile-induced MMs showed a significantly higher percentage of CD163-stained M2 macrophages than the crocidolite-induced tumors (*P* < 0.005, Fisher exact test). In addition, we stained serial MM sections of crocidolite-exposed and chrysotile-exposed *Bap1*-mutant mice with markers for arginase, which is considered a prototypic M2 macrophage marker. We also evaluated the expression of inducible nitric oxide synthase (iNOS), which is a functional marker of the M1 phenotype. As shown in Fig. 3B, arginase-1 (Arg-1) staining was more intense in MM tissue from chrysotile-induced MMs than in crocidolite-induced MMs, whereas iNOS staining was virtually absent in both tumor types and was mainly restricted to granulomatous lesions (Supplementary Fig. S5).

### Triple Indirect Sequential IF Staining Reveals a Predominance of M2 Versus M1 Macrophages in the TIME of MMs from *Bap1*-mutant Mice, Which is Even More Pronounced in Chrysotile-induced Than Crocidolite-induced Tumors

**FIGURE 4:**
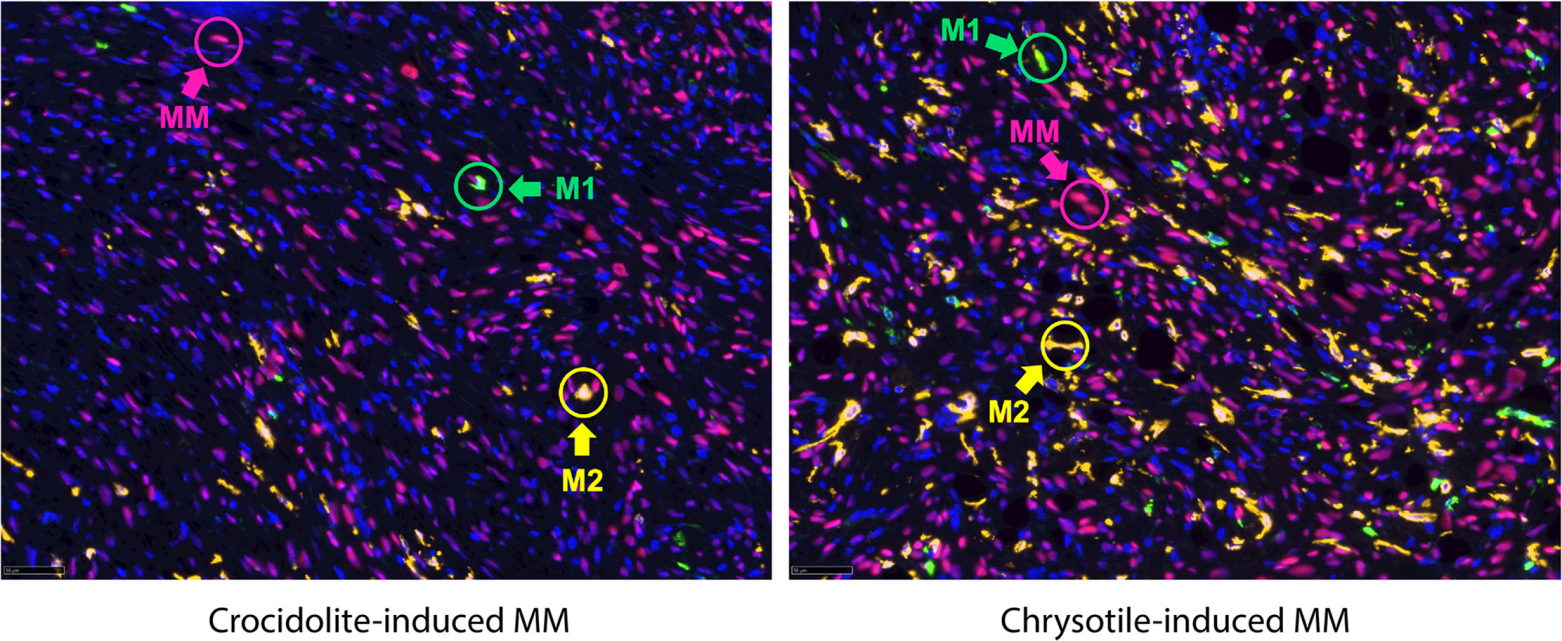
Triple indirect IF of crocidolite- and chrysotile-induced MMs from *Bap1^+/−^* mice. IF staining showing tumor cells as well as M1 and M2 macrophages in crocidolite- and chrysotile-induced MMs from 2 *Bap1^+/−^* mice. Histologic sections were subjected to triple indirect sequential IF using three different antibodies: WT1 to detect MM tumor cells (red circles; Red 555 fluorescence); CD11c to stain M1 macrophages (green circles; green 488 fluorescence); and CD163 marker to stain M2 macrophages (yellow circles; Cy5 yellow fluorescence).

Multiplex IF staining was performed to detect M1 and M2 macrophages in crocidolite- and chrysotile-treated MMs from *Bap1^+/−^* mice. Histologic sections were subjected to triple indirect sequential IF using WT1 to detect MM tumor cells, CD11c to identify M1 macrophages, and CD163 to identify M2 macrophages. These studies revealed a preponderance of M2 compared with M1 macrophages in the TIME of MMs from *Bap1^+/−^* mice, which was consistently more pronounced in chrysotile-induced MMs than in crocidolite-induced MMs (Fig. 4).

### Double IHC Staining Confirms a Prevalence of Cancer-associated Fibroblasts in Chrysotile-induced Compared with Crocidolite-induced MMs in *Bap1*-mutant Mice

Double IHC staining was performed on a set of chrysotile-induced and crocidolite-induced MMs from *Bap1^+/−^* mice. In addition to a greater presence of M2 macrophages in the TIME of chrysotile-induced MMs than in crocidolite-induced MMs, we observed an increased number of cancer-associated fibroblasts (CAF) in the TIME of chrysotile-induced MMs versus crocidolite-induced MMs (Supplementary Fig. S6). The overall greater numbers of CAFs in serpentine-induced MMs than in amphibole-induced MMs is consistent with the more profound protumorigenic immune response observed in chrysotile- than crocidolite-induced tumors.

### Expression of CD39 and C-C Motif Chemokine Receptor 2 are Elevated in Chrysotile-induced MMs Compared with Crocidolite-induced MMs from *Bap1*-mutant Mice

**FIGURE 5:**
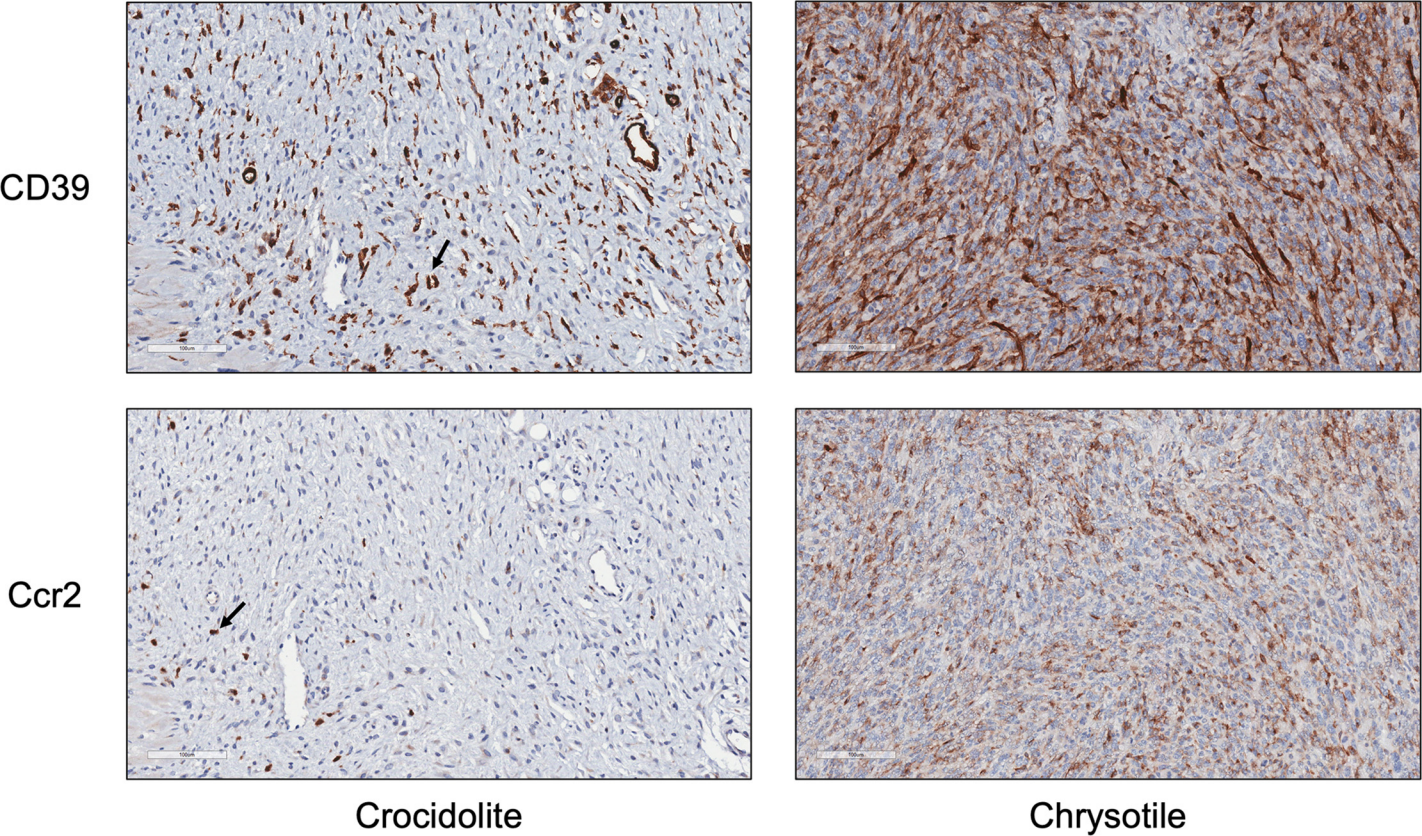
IHC staining for CD39 (top; diffuse brown stain) and Ccr2 (bottom; punctate stain) in crocidolite-induced and chrysotile-induced MMs from *Bap1^+/−^* mice. Arrows indicate examples of cells positively stained for Ccr2.

IL6 has been implicated in asbestos-induced MM, and IL6 release due to chronic inflammation in other cancers is known to promote the expression of CD39, the rate-limiting ecto-enzyme of the ATP-adenosine pathway. Because CD39/CD73-adenosine and C-C motif chemokine ligand 2 (Ccl2)/C-C motif chemokine receptor 2 (Ccr2) signaling are important pathways involved in creating an immunosuppressive TIME as well as tumor progression, we thus decided to evaluate the expression of CD39 and Ccr2 in peritoneal MMs induced by asbestos in *Bap1^+/^^−^* mice. IHC analyses revealed expression of both CD39 and Ccr2 in tumors from *Bap1-*mutant mice, with a markedly increased expression in chrysotile-induced MMs compared to that in crocidolite-induced MMs (Fig. 5).

### Expression of IL6 and IL10 are Elevated in Ascites From Chrysotile-exposed *Bap1^+/^^−^* Mice with MM Compared with That in Ascites from Chrysotile-exposed *Bap1^+/+^* Mice with MM

**FIGURE 6:**
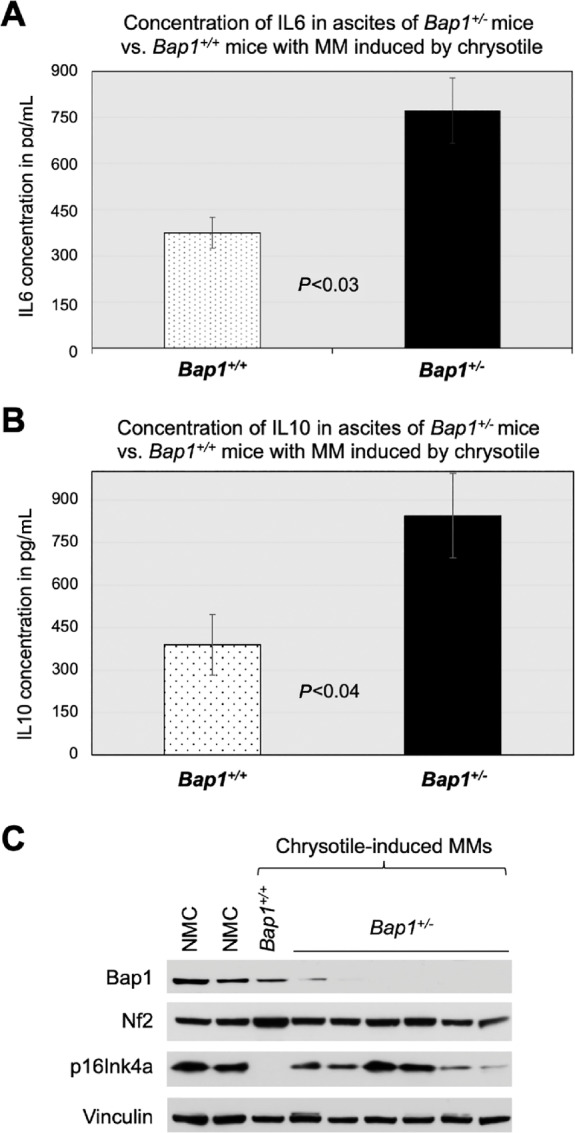
Expression of IL6 and IL10 in ascites and Bap1 and p16^Ink4a^ in MM tumors from chrysotile-exposed *Bap1^+/^^−^* mice. **A,** Concentration of IL6 in ascites of *Bap1^+/^^−^* mice compared with that from *Bap1^+/+^* mice with MMs induced by chrysotile. Ascitic fluids were obtained from 5 *Bap^+/+^* mice and 4 *Bap1^+/^^−^* mice with chrysotile-induced MMs. **B,** Expression of IL10 in ascites of 4 *Bap1^+/^^−^* mice versus that in ascites of 5 *Bap1^+/+^* mice with MMs induced by chrysotile. **C,** Immunoblot analysis of tumor suppressors Bap1, Nf2, and p16Ink4a in MM tumors from 1 chrysotile-exposed *Bap1^+/+^* mouse and 6 chrysotile-exposed *Bap1*^+/−^ mice. NMC, normal (mouse) mesothelial cells; Vinculin, loading control.

Because IL6 cooperates synergistically with Ccl2/Ccr2 signaling to alter the TIME toward immunosuppression by attracting M2 macrophages and activating associated factors (19), we evaluated the expression of IL6 in ascitic fluids from *Bap1^+/^^−^* and WT mice that developed chrysotile-induced MMs using an IL6 ELISA. Ascites from *Bap1^+/^^−^* mice showed significantly elevated expression of IL6 compared with that observed in WT mice (Fig. 6A). IL10, like IL6, is an immunosuppressive factor that can induce expression of CD163 through the STAT signaling pathway and is expressed at high levels in M2 macrophages (20). Thus, we also decided to evaluate the levels of IL10 in ascitic fluids from *Bap1^+/^^−^* and WT mice that developed chrysotile-induced MMs, using an IL10 ELISA. As with IL6, ascites from *Bap1^+/^^−^* mice showed significantly elevated expression of IL10 compared with that in WT mice (Fig. 6B).

### Immunoblot Analyses Demonstrate That Chrysotile-induced MMs from *Bap1*^+/−^ Mice Show Loss of Bap1 Expression

Immunoblot analysis was performed on MM tumors from mice exposed to low-dose (0.4 mg/mouse) chrysotile. In a single MM tumor from a *Bap1^+/+^* mouse with sufficient specimen available for this analysis, there was expression of Bap1 protein but loss of detectable p16Ink4a. In contrast, in six MMs from chrysotile-exposed *Bap1^+/^^−^* mice, immunoblotting demonstrated loss or greatly reduced expression of Bap1 and retention of p16Ink4a expression (Fig. 6C). This reciprocal pattern is identical to that we previously reported in MMs from crocidolite-exposed *Bap1^+/^^−^* and *Bap1^+/+^* mice (11). Nf2/merlin expression was retained in all the specimens tested.

### Preliminary Clinical Connections

**FIGURE 7:**
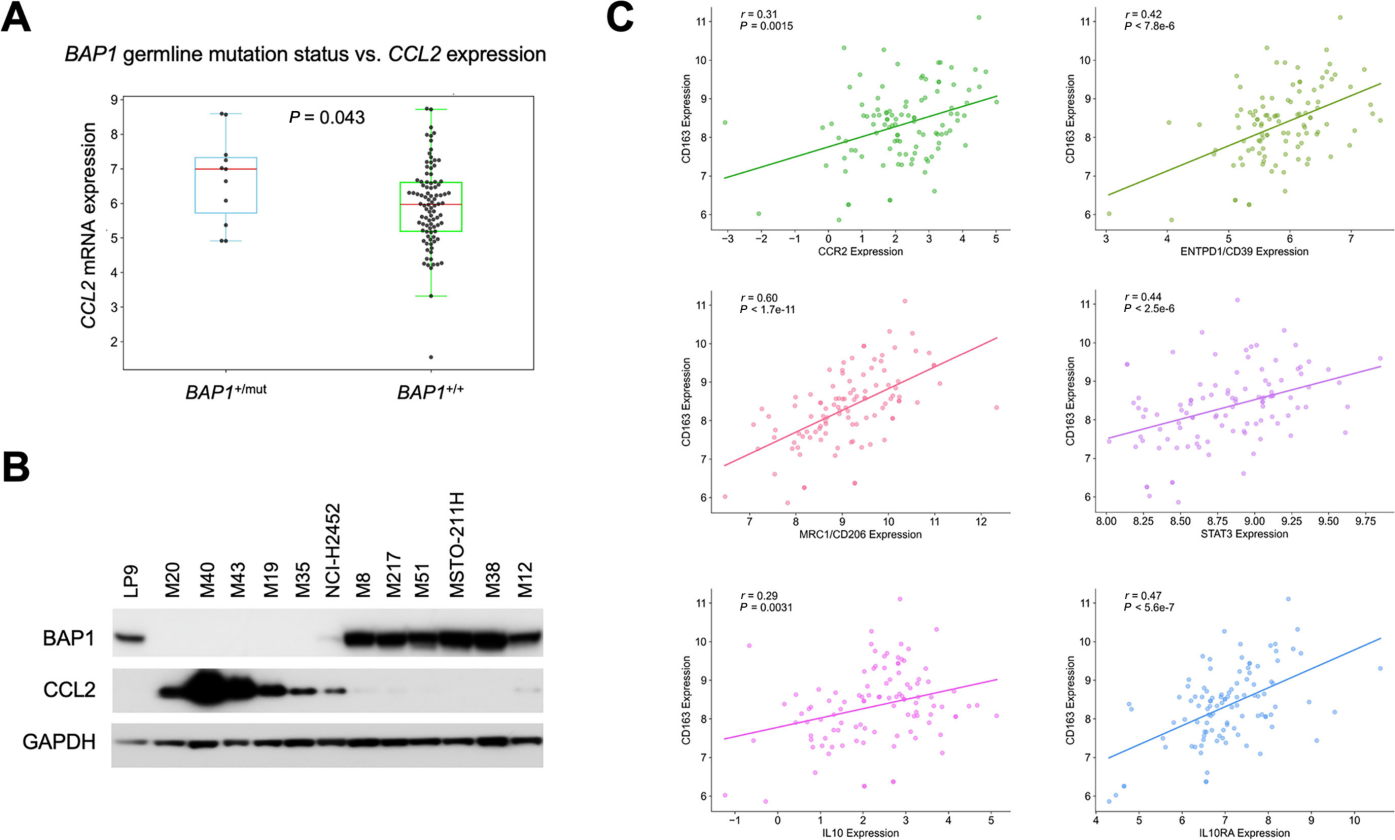
Preliminary clinical connections of mouse findings based on NCI human MM RNA-seq and germline mutation data (16). **A,** Comparison of *CCL2* mRNA expression levels based on *BAP1* germline mutation status in 100 MM samples from the NCI cohort. Statistical analysis was performed using the Student *t* test. **B,** Immunoblot analyses of CCL2 in human MM cell lines with or without loss of BAP1 expression. GAPDH was used as a loading control. **C,** Scatter plots depicting the relationship between *CD163* and *CCR2*, *ENTPD1* (CD39), *IL10*, *IL10RA*, *MRC1* (CD206), and *STAT3* mRNA expression levels. Statistical analysis for this correlation study was conducted using Spearman rank-order correlations (*r*) and *P* values.

To assess the clinical relevance of our mouse model findings, we analyzed NCI human MM RNA-seq data (*n* = 100) and germline variants data (16). We examined clinical and pathologic aspects of sporadic MMs and MM tumors from patients with germline *BAP1* mutations (*n* = 11), investigating gene expression correlations between genes related to M1/M2 macrophages and other immune signatures outlined in our investigation. Interestingly, we found that tumors from patients with MM with germline *BAP1* mutations displayed a significantly higher expression level of *CCL2* compared with that in tumors from sporadic MM cases without germline *BAP1* mutations (*P* = 0.043; Fig. 7A). Immunoblot analyses of a set of human MM cell lines similarly showed that cell lines with loss of BAP1 exhibited high levels of CCL2 expression, whereas MM cell lines that retained expression of BAP1 had little or no expression of CCL2 (Fig. 7B). Moreover, in the entire 100 NCI human MM RNA-seq dataset (16), *CD163* mRNA expression showed a highly significant positive correlation with multiple genes encoding proteins involved in the CD39/CD73–adenosine, CCR2, and other immunosuppressive pathways, that is, *IL10*, *IL10RA*, *MRC1* (CD206), and *STAT3* (Fig. 7C), forming an interconnected network consistent with our mouse data.

## Discussion

The work presented here demonstrates that chronic intraperitoneal exposure to either crocidolite or chrysotile can readily induce MM in *Bap1*-mutant mice. In fact, for chrysotile, a higher incidence of MM was observed at the lower total dose used (0.4 mg/mouse) than at 3.2 mg/mouse, although the time to tumor onset was much longer. It is likely that the higher dose of chrysotile produced an excessive amount of inflammation, resulting in some animals succumbing to adhesion-related intestinal obstructions and/or liver fibrosis before the MM could develop. As shown in Supplementary Table S2, these fibrotic pathologies occurred in *Bap1^+/+^* and *Bap1^+/^^−^* mice at similar rates at all doses of asbestos tested. However, approximately 30% more animals developed liver fibrosis and/or other adhesions when exposed to the higher total dose of chrysotile (3.2 mg) than at the lower dose (0.4 mg).

While the peritoneal space is not the usual site of exposure to asbestos in humans, it is used for almost all asbestos carcinogenicity studies in mice (21). This is because unlike rats, mice have a comparatively small pleural space for inoculation and has an ill-suited nasal passage architecture for asbestos inhalation studies (21).

In humans, asbestos fibers can enter the peritoneal space by penetrating the walls of the alimentary canal. The fact that chrysotile can be cleared from the lungs, coughed up, and swallowed provides an opportunity for these fibers to enter the alimentary canal, with a fraction of the fibers gaining entry into the peritoneal cavity. Whether this has relevance to the fact that *BAP1* mutation carriers develop a disproportionate number of peritoneal MMs is not known, but it is notable that in the BAP1-TPDS, the peritoneal form of MM predominates (9), which is in stark contrast to the sporadic disease, where peritoneal MM accounts for only 10%–15% of cases (22).

Using a different *Bap1^+/^^−^* mouse model, Napolitano and colleagues reported that minimal exposure to crocidolite was associated with alterations in the peritoneal inflammatory response, including elevated levels of M2 macrophages (12). Our data indicate that this association with M2 macrophages is much greater in chrysotile-exposed than in crocidolite-exposed *Bap1^+/^^−^* mice.

The immune system plays a major role in the pathogenesis, prognosis, and treatment of MM. Immunotherapeutic approaches for MM have been hampered because many of these tumors have an immunosuppressed TIME. In recent years, strategies designed to activate the innate immunity of the TIME have emerged in an effort to turn “cold” tumors “hot” (23). In malignant pleural mesothelioma (MPM), pleural effusion (PE) occurs in approximately 70% of cases and consists of tumor cells and various types of immune and stromal cells (24). Immune cells invade both the tumor and PE in patients with MPM, and TAMs are a major component of the TIME in patients with MPM (25). Depending on the specific stimuli within the TIME, TAMs can develop into a tumor-inhibitory (M1) or tumor-promoting (M2) phenotype (26). Moreover, the presence of M2 macrophages in human MPM biopsies is associated with poor survival (25). In our mouse model, IHC and IF findings with multiple immune cell markers revealed an abundance of M2 macrophages infiltrating these tumors, whereas CD3-positive T cells, B cells, and NK cells were depleted. In contrast, proinflammatory M1 macrophages predominated in granulomatous lesions. This was expected given that granulomas are, by definition, noncancerous, small areas of inflammation.

A consistently higher percentage of CD163^+^ M2 macrophages and a marked increase of Arg-1 staining were seen in the TIME of chrysotile-induced than crocidolite-induced MMs. This is important because CD163^+^ macrophages are particularly immunosuppressive and linked to a poor prognosis (27). Thus, the generally shorter survival observed in chrysotile-exposed versus crocidolite-exposed *Bap1^+/^^−^* mice appears to correlate with poorer survival in patients with MM whose tumors harbor M2 macrophages (25). Interestingly, CD163 is a novel target gene of STAT3 and has been proposed as a therapeutic target in cancer (28).

The primary function of M2-type macrophages is protumorigenic via cross-talk with other elements of the TIME and tumor cells. Cross-talk between tumor cells and TAMs is mediated by Ccl2/Ccr2 expression in the TIME, which is responsible for remodeling of the extracellular matrix, cancer progression, evasion of immunosurveillance, and angiogenesis. When mesothelial cells encounter asbestos, a cascade of inflammatory reactions begins, including the secretion of Ccl2, IL1β, and IL6. In our chrysotile-induced mouse MMs, an ELISA revealed increased levels of IL6 in the ascitic fluids of *Bap1^+/^^−^* mice compared with those from WT littermates. Furthermore, IHC analysis of MMs from *Bap1^+/^^−^* mice demonstrated upregulation of Ccr2 in tumors from chrysotile-exposed versus crocidolite-exposed animals. IL6, one of the major players in asbestos-induced chronic inflammation implicated in MM (29), can cooperate synergistically with Ccl2/Ccr2 signaling to alter the TIME toward immunosuppression by attracting myeloid-derived suppressor cells (MDSC), M2 macrophages, and regulatory T cells (Treg) as well as activating associated factors (19). Moreover, high concentrations of IL6 have been reported in PEs from patients with MPM, with production found to arise specifically from malignant cells (30). In addition, IL6 release due to chronic inflammation is known to induce the expression of CD39, and IL6-induced CD39 expression on tumor-infiltrating NK cells has been reported to predict poor prognosis in esophageal squamous cell carcinoma (31). To further define the TIME in peritoneal MMs induced by asbestos, we evaluated the expression of CD39, the rate-limiting ecto-enzyme of the ATP-adenosine pathway. The rationale for doing so was that the CD39/CD73-adenosine pathway is known to contribute to an important tumor-induced immunosuppressive mechanism. In our *Bap1*-mutant model, we observed a marked increase in the expression of CD39 in chrysotile-induced MMs compared with crocidolite-induced MMs. In cancers, including MM, inflammatory and hypoxic conditions lead to overexpression of the adenosine production machinery, such as CD39 and CD73 (ecto-5′-nucleotidase) in tumor cells, CAFs, Tregs, and MDSCs, which consequently results in an excessive accumulation of extracellular adenosine (32, 33). Increased amounts of extracellular adenosine are responsible for the creation of an immunosuppressive TIME as well as tumor progression and evasion (34). The accumulation of adenosine suppresses cytokine production and the proliferation of CD8^+^ T cells as well as the cytotoxic CD8^+^ T-cell activity responsible for anti-tumor responses in the TIME. Thus, targeting the CD39/CD73-adenosine pathway together with the use of an immune checkpoint inhibitor could prove to be therapeutically efficacious in various cancers, including MM.

M2 macrophages secrete abundant amounts of IL10 as well as IL6. IL10 is a multifaceted immune-suppressive cytokine that suppresses cytotoxicity mediated by antitumorigenic NK cells and cytotoxic T cells. IL10 promotes differentiation of Tregs, enhances tumor cell survival, proliferation, and metastasis by controlling antitumor immunity mostly through the STAT3 signaling pathway via the IL10 receptor (IL10R), whereas IL6 activates Th2 cells to promote tumor progression (35). Furthermore, TAM-secreted IL10 has been shown to drive an immunoevasive microenvironment associated with a poor prognosis and inferior therapeutic response to adjuvant chemotherapy (36). In our chrysotile-exposed mice that developed MM, expression of IL10 was significantly elevated in the ascitic fluids of *Bap1^+/^^−^* mice compared with those from WT littermates, consistent with the susceptibility of *Bap1*-mutant mice to chrysotile-induced immune suppression.

In conclusion, our collective findings indicate that chrysotile induces a profound inflammatory immune response in germline *Bap1* heterozygous mice even at low doses. We demonstrate that chrysotile as well as crocidolite can be carcinogenic in our *in vivo* model. Our findings also show that chrysotile-induced inflammation results in an immunosuppressive tumor microenvironment that creates favorable conditions permitting MM cells to evade immune surveillance. This may have considerable relevance at the current time because chrysotile now makes up approximately 95% of asbestos used commercially. While our results in WT mice indicate that minimal exposure to both crocidolite and chrysotile causes comparatively few MMs, mice carrying a germline mutation in *Bap1* are at very high risk of MM. Drawing parallels to human disease, our experimental findings indicate that *BAP1* mutation carriers are highly susceptible to the carcinogenic effects of even minimal amounts of asbestos, including both amphibole and serpentine forms. The robust generation of M2 macrophages, particularly the CD163^+^ subpopulation, and CAFs by asbestos contributes to an immunosuppressive TIME in *Bap1*-mutant mice, suggesting that chemoprevention or immunotherapeutic strategies targeting CD163^+^ TAMs, CD39/CD73-adenosine, and Ccl2/Ccr2−IL6/IL10 signaling might reprogram MM from a tumor-promoting “M2-like” phenotype to a tumoricidal “M1-like” phenotype, potentially benefitting *BAP1* mutation carriers and others with germline pathogenic mutations. The fact that mRNA profiling data in human sporadic MMs and MM carriers of germline *BAP1* mutations show immune signatures similar to those observed in our mouse model suggest that the findings presented here have clinical relevance and potential therapeutic implications.

## Supplementary Material

Supplementary Table S1 Primary antibodies used for IHC, IF, and immunoblot analyses

Supplementary Table S2 Summary of the ratio of asbestos-exposed mice in each cohort that developed liver fibrosis and/or other abdominal adhesions

Supplementary Figure S1 Anatomical image of peritoneal MMs (white long arrows) induced by chronic intraperitoneal injections of chrysotile in a Bap1+/- mouse. MMs developing in Bap1+/- and Bap1+/+ (wild type) littermates injected with either chrysotile or crocidolite generally were diffuse peritoneal lesions sometimes accompanied by ascites. Note that the MMs shown in this animal are unusually large for illustrative purposes. Most tumors in this model were solitary, much smaller, and diffuse. Irregular liver surface (yellow rectangle) is likely indicative of fibrosis. L, liver; LI, large intestine; M, mesothelioma.

Supplementary Figure S2 H&E images of liver fibrosis in Bap1+/- mice chronically injected i.p. with asbestos. A) Interlobular liver fibrosis in a chrysotile-exposed mouse. B) Liver fibrosis in between liver and spleen in chrysotile-injected mouse. C) Fibrosis on surface of liver in a crocidolite-exposed mouse. D) Fibrosis between liver and pancreas of a crocidolite-exposed mouse. All original images are at 200X magnification. Abbreviations: F, fibrosis; L, liver; M, mesothelioma; S, spleen; P, pancreas.

Supplementary Figure S3 Histopathologic assessment of two representative MMs from Bap1+/− mice exposed to either crocidolite or chrysotile. Serial tumor sections depicting H&E staining and IHC for MM markers podoplanin and WT1 in a representative crocidolite-induced MM (upper panel) and a representative chrysotile-induced MM (lower panel). All original images are at 200X magnification.

Supplementary Figure S4 Tumor immune microenvironment of chrysotile-induced MM from a Bap1+/- mouse. Serial sections of MM stained with several different immune cell markers: CD3 (T cells), CD45R (B cells), F4/80 (macrophages), and NK1.1 (natural killer cells), clockwise starting from upper left. T, B, and NK cells are depleted in tumor, whereas numerous cells in and around the tumor are stained with the macrophage marker F4/80.

Supplementary Figure S5 Expression of arginase and iNOS in a crocidolite-exposed Bap1+/- mouse. Arginase-1 (Arg-1) staining is found mostly in mesothelioma tissue (MM), whereas iNOS staining is present primarily in the granulomatous lesions (G). Arrows indicate crocidolite fibers found in the granuloma.

Supplementary Figure S6 IHC staining for WT1 (purple) and SMA (brown in top panels; teal in bottom panels) in representative crocidolite-induced and chrysotile-induced MMs from Bap1+/- mice. WT1 stains MM cells, whereas SMA stains CAFs, the latter typically more abundant in chrysotile-induced than crocidolite-induced MMs, as in the examples shown here.
