# Supplementary material for: Low Exposures to Amphibole or Serpentine Asbestos in Germline Bap1-mutant Mice Induce Mesothelioma Characterized by an Immunosuppressive Tumor Microenvironment
Source: Cancer Res Commun. 2024 Apr 8;4(4):1004–15. doi: 10.1158/2767-9764.CRC-23-0423 (PMC11000687; doi:10.1158/2767-9764.CRC-23-0423)
Supplement: Supplementary Table S1 — Primary antibodies used for IHC, IF, and immunoblot analyses [file crc-23-0423-s01.pdf]

Supplementary Table S1. Primary antibodies used for IHC, IF, and immunoblot analyses

Primary Antibodies	RRID	Dilution	Company	Clone/Cat. No.
anti-Arginase-1	AB_2800207	1:300	Cell Signaling	D4E3M, rabbit mAb #93668
anti-Bap1	AB_2798168	1:2000	Cell Signaling	D7W7O, rabbit mAb #13271
anti-CCR2 antibody	AB_2893307	1:100	Abcam	EPR2084415, rabbit mAb #ab273050
anti-CD3ε	AB_2755035	1:50	Cell Signaling	D4V8L,rabbit mAb #99940
anti-CD11c	AB_2924836	1:100	Cell Signaling	D1V9Y, rabbit mAb #39143
anti-CD39	AB_2889212	1:1000	Abcam	EPR20627, rabbit mAb #ab223842
anti-CD45R/B220	AB_394616	1:100	BD Biosciences	Clone RA3-6B2, Biotin Conjugated, rat mAb #553086
anti-CD163	AB_2753196	1:200	Abcam	EPR19518, rabbit mAb #ab182422
anti-CD206	AB_2892682	1:600	Cell Signaling	E6T5J, rabbit mAb #24595
anti-F4/80	AB_2799771	1:800	Cell Signaling	D2S9R, rabbit mAb #70076
anti-iNOS	AB_3083470	1:50	Abcam	RM1017, rabbit mAb #ab283655
anti-Nf2	AB_2798056	1:4000	Cell Signaling	D6N8H, rabbit mAb #12896
anti-NK1.1	AB_2296673	1:50	Thermo Fisher Scientific	PK136, mouse mAb #MA1-70100
anti-p16Ink4a	AB_2891084	1:2000	Abcam	EPR20418, rabbit mAb #ab211542
anti-Podoplanin	AB_298718	1:100	Abcam	RTD4E10, Syrian hamster mAb #ab11936
anti-SMA	AB_3065058	1:100	BioGenex	HHF35, mouse mAb #AM090-GP
anti-Vinculin	AB_2728768	1:50,000	Cell Signaling	E1E9V, rabbit mAb #13901
anti-WT1	AB_2043201	1:30	Abcam	CAN-R9 (IHC)-56-2, rabbit mAb #ab89901
anti-MCP1 (CCL2)	AB_3083804	1:1000	Abcam	EPR21025, rabbit mAb #ab214819
Anti-GAPDH	AB_10622025	1:50000	Cell Signaling	D16H11 XP, rabbit mAb #5174
