# Supplementary material for: Low Exposures to Amphibole or Serpentine Asbestos in Germline Bap1-mutant Mice Induce Mesothelioma Characterized by an Immunosuppressive Tumor Microenvironment
Source: Cancer Res Commun. 2024 Apr 8;4(4):1004–15. doi: 10.1158/2767-9764.CRC-23-0423 (PMC11000687; doi:10.1158/2767-9764.CRC-23-0423)
Supplement: Supplementary Table S2 — Summary of the ratio of asbestos-exposed mice in each cohort that developed liver fibrosis and/or other abdominal adhesions [file crc-23-0423-s02.pdf]

Supplementary Table S2. Summary of the ratio of asbestos-exposed mice in each cohort that developed liver fibrosis and/or other abdominal adhesions[1]

Genotype	Crocidolite	Chrysotile	
	Total dose = 0.8 mg	Total dose = 3.2 mg	Total dose = 0.4 mg
Bap1 $^{+/-}$	13/20 (65%)	22/26 (85%)	10/18 (56%)
Bap1 $^{+/+}$	13/22 (59%)	20/23 (87%)	9/15 (60%)

[1]Many of the mice with fibrosis and/or other abdominal lesions also had peritoneal malignant mesothelioma (MM).
