# Supplementary figures and images for: Low Exposures to Amphibole or Serpentine Asbestos in Germline Bap1-mutant Mice Induce Mesothelioma Characterized by an Immunosuppressive Tumor Microenvironment
Source: Cancer Res Commun. 2024 Apr 8;4(4):1004–15. doi: 10.1158/2767-9764.CRC-23-0423 (PMC11000687; doi:10.1158/2767-9764.CRC-23-0423)

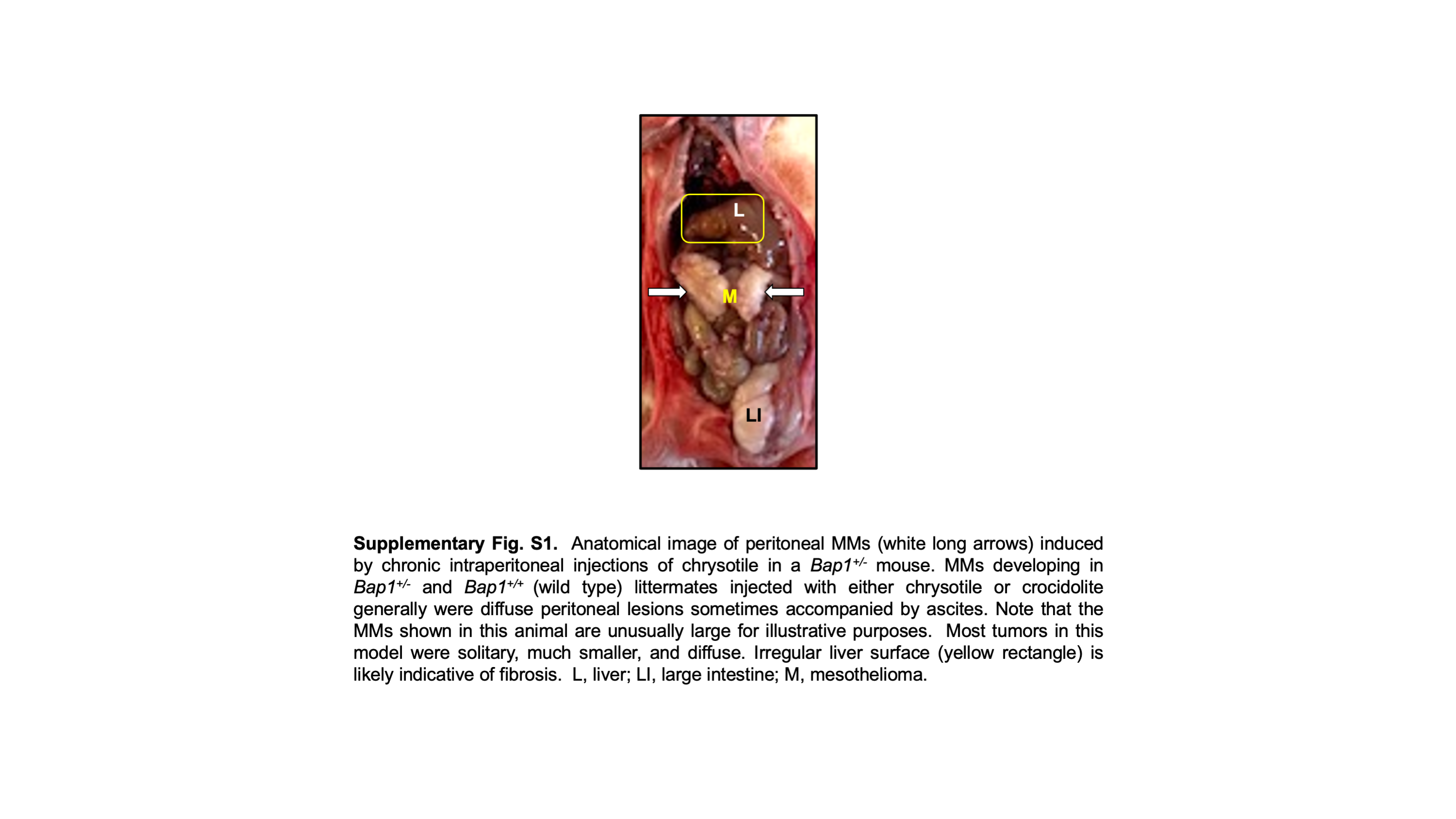

Supplement: Supplementary Figure S1 — Anatomical image of peritoneal MMs (white long arrows) induced by chronic intraperitoneal injections of chrysotile in a Bap1+/- mouse. MMs developing in Bap1+/- and Bap1+/+ (wild type) littermates injected with either chrysotile or crocidolite generally were diffuse peritoneal lesions sometimes accompanied by ascites. Note that the MMs shown in this animal are unusually large for illustrative purposes. Most tumors in this model were solitary, much smaller, and diffuse. Irregular liver surface (yellow rectangle) is likely indicative of fibrosis. L, liver; LI, large intestine; M, mesothelioma. [file crc-23-0423-s03.png]

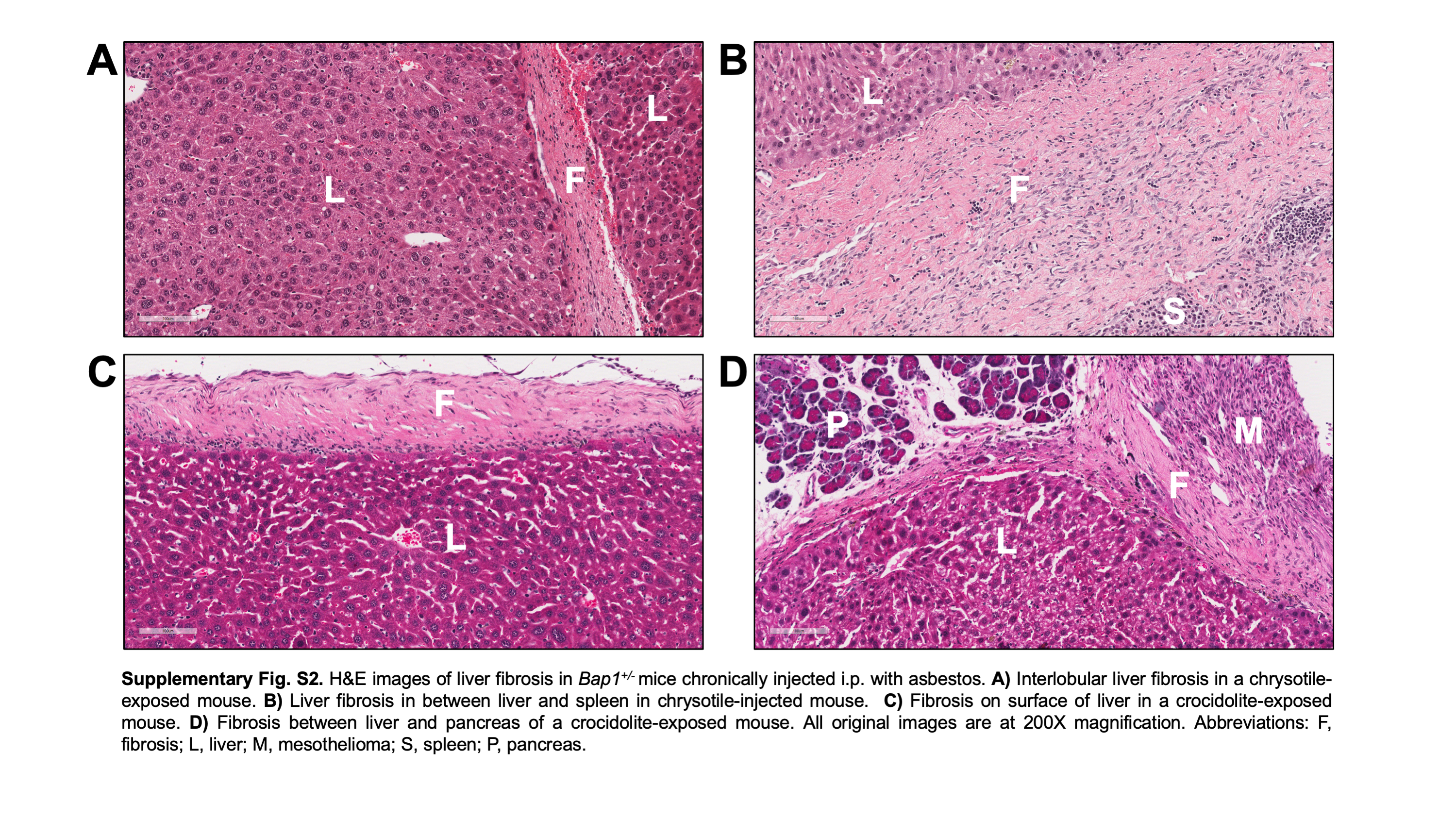

Supplement: Supplementary Figure S2 — H&E images of liver fibrosis in Bap1+/- mice chronically injected i.p. with asbestos. A) Interlobular liver fibrosis in a chrysotile-exposed mouse. B) Liver fibrosis in between liver and spleen in chrysotile-injected mouse. C) Fibrosis on surface of liver in a crocidolite-exposed mouse. D) Fibrosis between liver and pancreas of a crocidolite-exposed mouse. All original images are at 200X magnification. Abbreviations: F, fibrosis; L, liver; M, mesothelioma; S, spleen; P, pancreas. [file crc-23-0423-s04.png]

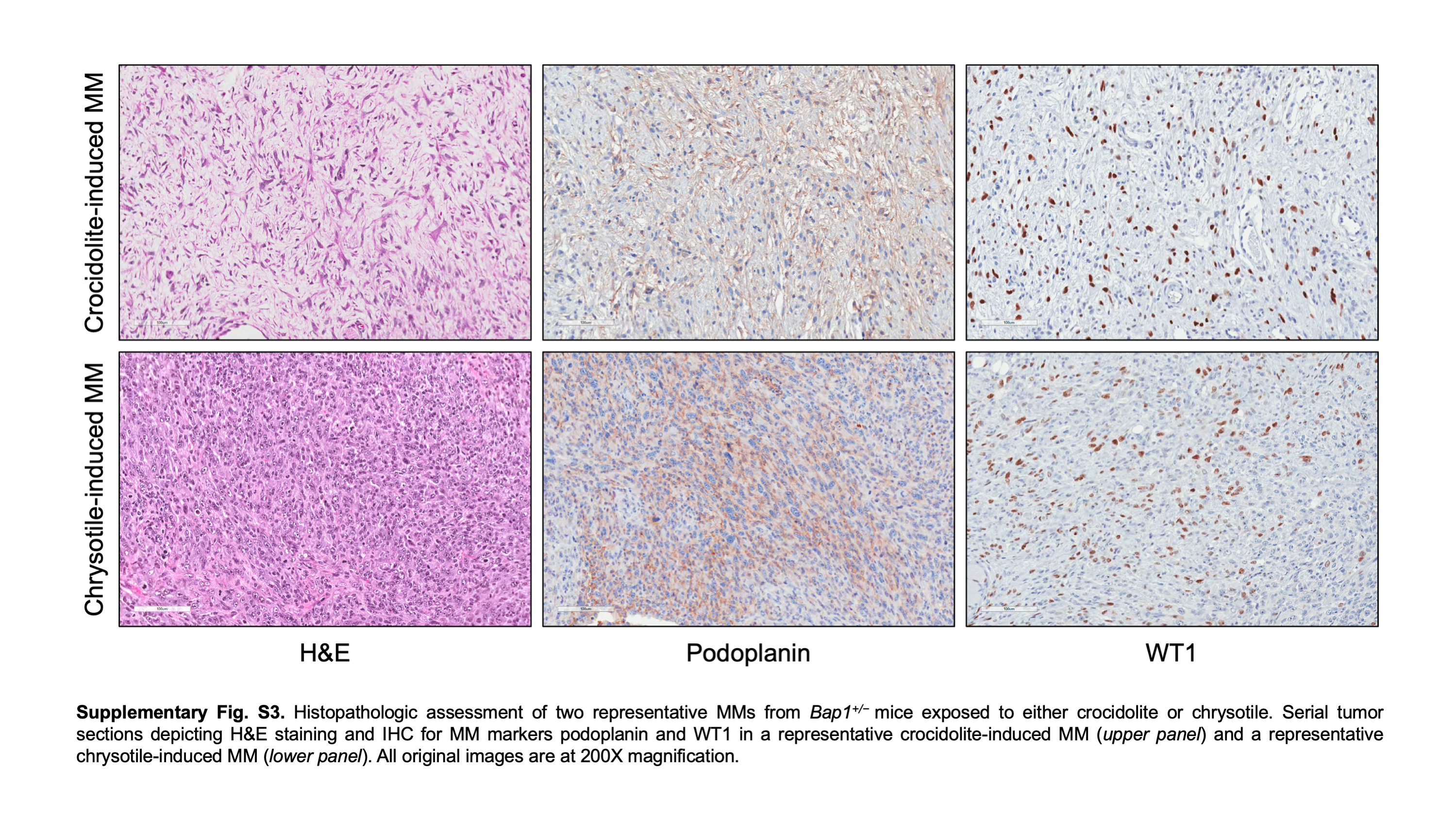

Supplement: Supplementary Figure S3 — Histopathologic assessment of two representative MMs from Bap1+/− mice exposed to either crocidolite or chrysotile. Serial tumor sections depicting H&E staining and IHC for MM markers podoplanin and WT1 in a representative crocidolite-induced MM (upper panel) and a representative chrysotile-induced MM (lower panel). All original images are at 200X magnification. [file crc-23-0423-s05.png]

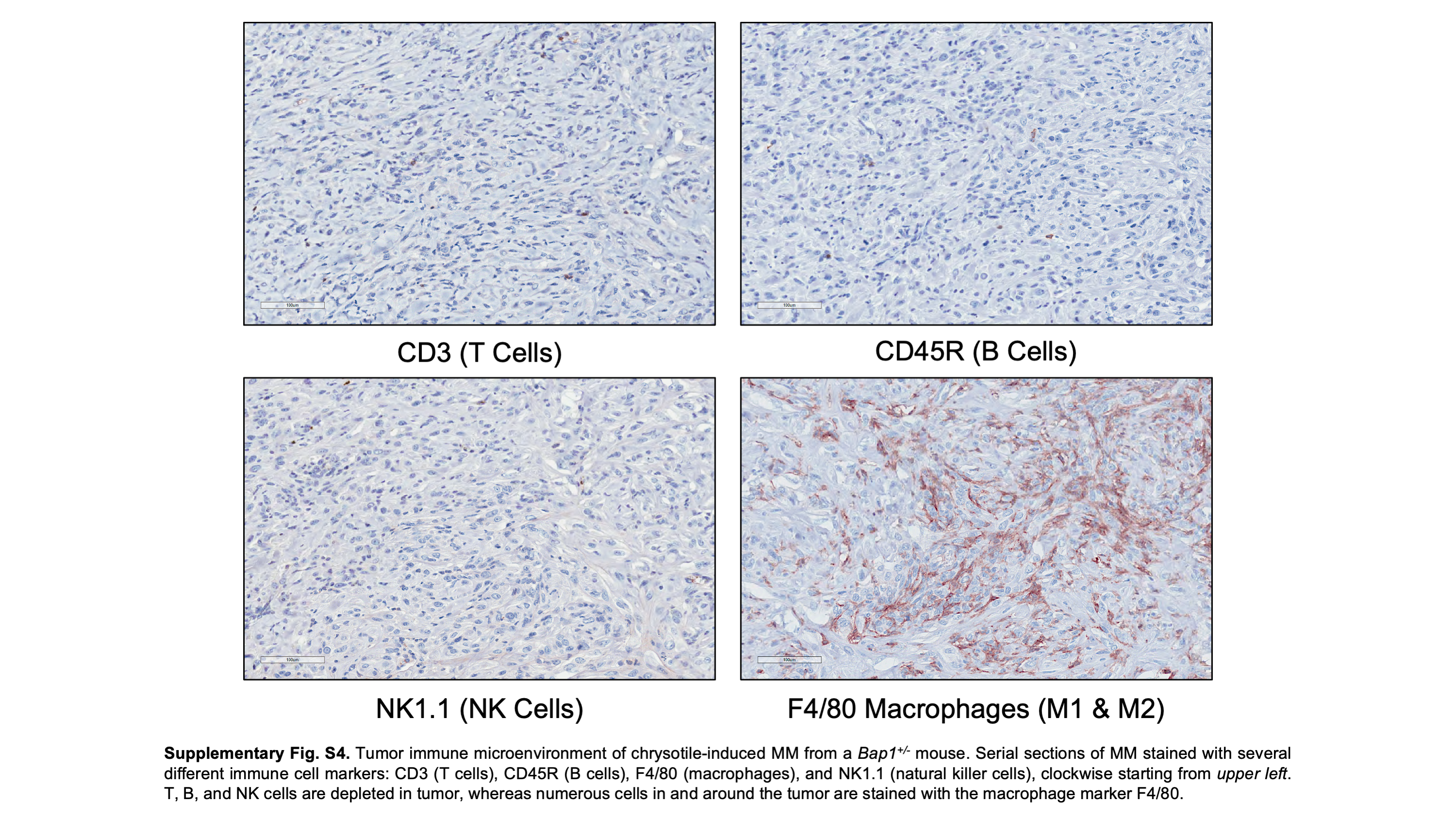

Supplement: Supplementary Figure S4 — Tumor immune microenvironment of chrysotile-induced MM from a Bap1+/- mouse. Serial sections of MM stained with several different immune cell markers: CD3 (T cells), CD45R (B cells), F4/80 (macrophages), and NK1.1 (natural killer cells), clockwise starting from upper left. T, B, and NK cells are depleted in tumor, whereas numerous cells in and around the tumor are stained with the macrophage marker F4/80. [file crc-23-0423-s06.png]

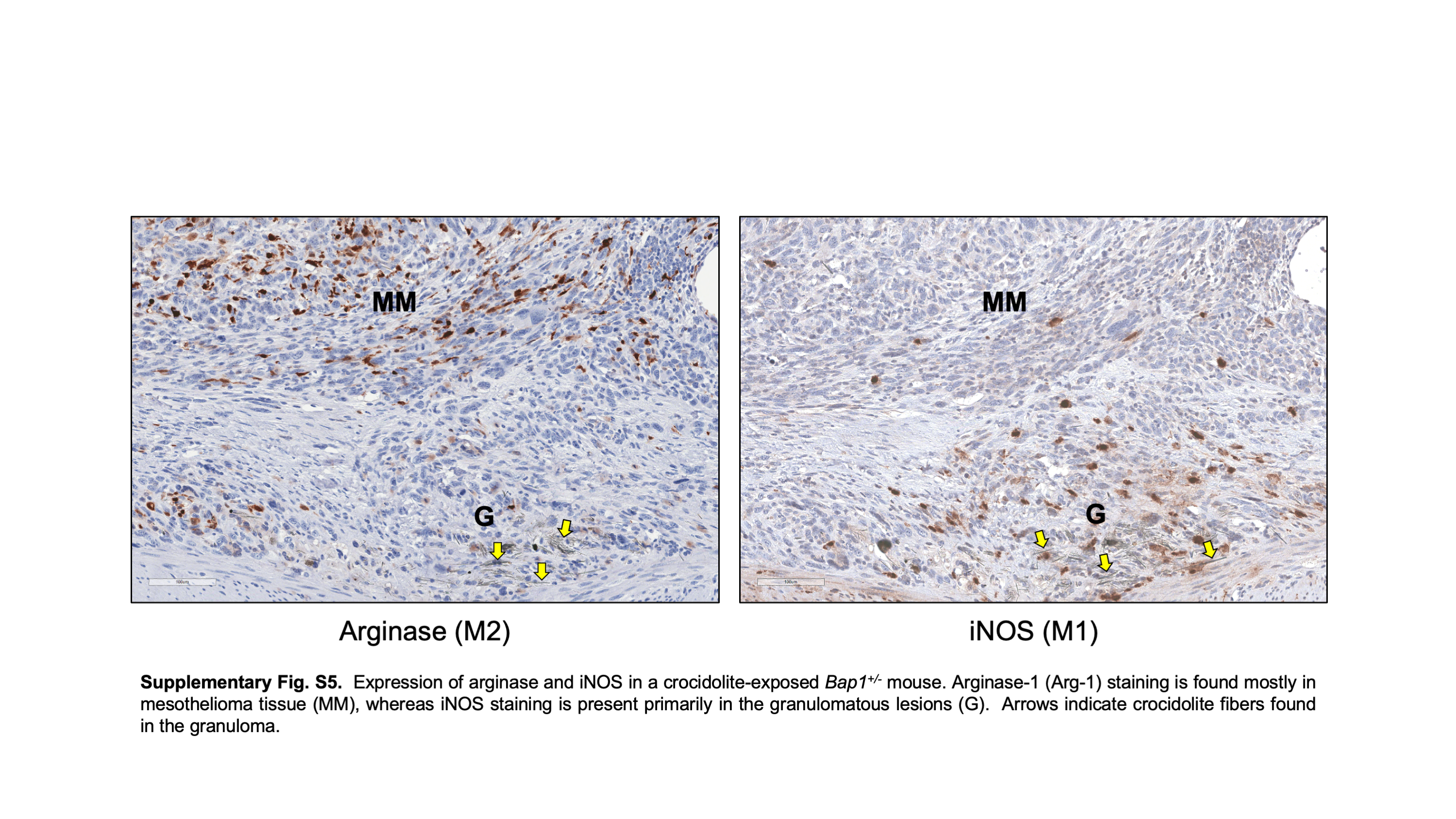

Supplement: Supplementary Figure S5 — Expression of arginase and iNOS in a crocidolite-exposed Bap1+/- mouse. Arginase-1 (Arg-1) staining is found mostly in mesothelioma tissue (MM), whereas iNOS staining is present primarily in the granulomatous lesions (G). Arrows indicate crocidolite fibers found in the granuloma. [file crc-23-0423-s07.png]

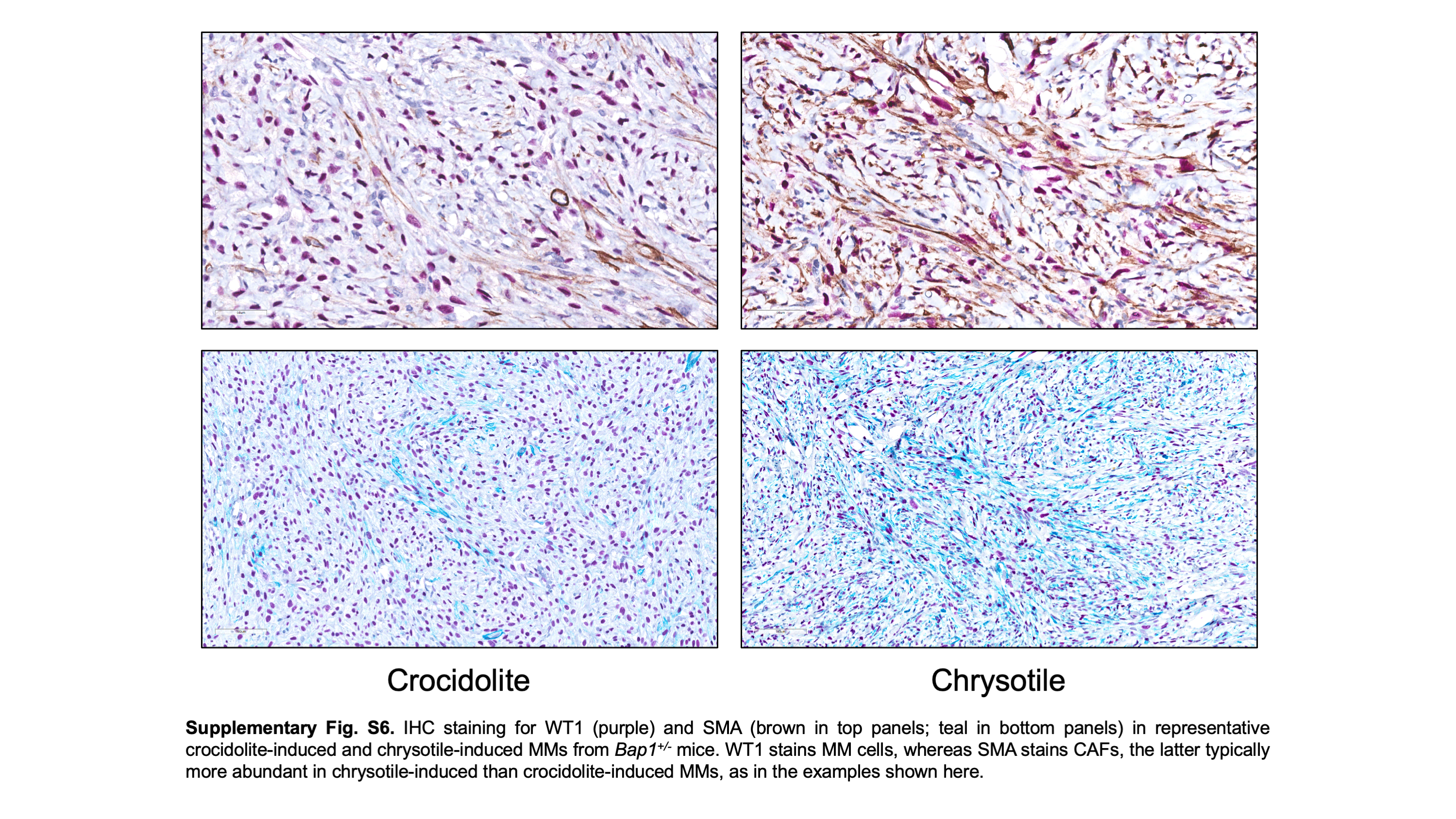

Supplement: Supplementary Figure S6 — IHC staining for WT1 (purple) and SMA (brown in top panels; teal in bottom panels) in representative crocidolite-induced and chrysotile-induced MMs from Bap1+/- mice. WT1 stains MM cells, whereas SMA stains CAFs, the latter typically more abundant in chrysotile-induced than crocidolite-induced MMs, as in the examples shown here. [file crc-23-0423-s08.png]
